# Trends in the incidence and prevalence of Parkinson’s disease in Korea: a nationwide, population-based study

**DOI:** 10.1186/s12877-019-1332-7

**Published:** 2019-11-21

**Authors:** Joo-Hyun Park, Do-Hoon Kim, Do-Young Kwon, Moonyoung Choi, Shinhye Kim, Jin-Hyung Jung, Kyungdo Han, Yong-Gyu Park

**Affiliations:** 1 Department of Family Medicine, Korea University Ansan Hospital, Korea University College of Medicine, 123, Jeokgeum-ro, Danwon-gu, Ansan-si, Gyeonggi-do 15355 Republic of Korea; 2 Department of Neurology, Korea University Ansan Hospital, Korea University College of Medicine, 123, Jeokgeum-ro, Danwon-gu, Ansan-si, Gyeonggi-do 15355 Republic of Korea; 3 Department of Family Medicine, Gangnam Severance Hospital, Yonsei University College of Medicine, Seoul, Republic of Korea; 4 Department of Biostatics, Catholic University College of Medicine, Seoul, Republic of Korea

**Keywords:** Parkinson’s disease, Incidence, Prevalence, Epidemiology, Neurodegenerative disease

## Abstract

**Background:**

The lack of adequate and detailed epidemiological data of Parkinson’s disease (PD), especially in Asia, is a barrier to future disease burdens and the prospect of effective public health plans. This study aimed to investigate temporal trends in the incidence and prevalence of PD in South Korea from 2010 to 2015, based on uniform diagnostic criteria.

**Methods:**

This study examined all PD patients registered in a South Korean national registry database of more than 50 million individuals. We analyzed the incidence and prevalence of PD according to age, gender, and region.

**Results:**

The annual incidence of PD was between 22.4–27.8 cases per 100,000 individuals. During the 6-year study period, there were 73,726 new PD patients, 42.3% of whom were men. The standardized incidence of PD increased over time in men but remained constant in women until 2013 but began to increase in 2014. The female-to-male ratio in the incidence of PD was 1.4:1 while the female-to-male ratio in the prevalence of PD was 1.6:1. The age- and gender-standardized prevalence of PD increased from 115.9 cases per 100,000 individuals in 2010 to 139.8 cases per 100,000 individuals in 2015. From 2014, the incidence and prevalence of PD peaked in individuals aged between 80 and 89 years in both men and women. Regional analysis also showed an increased prevalence of PD in all regions of Korea.

**Conclusions:**

The incidence and prevalence of PD in Korea were higher in women and increased gradually from 2010 to 2015. The findings may contribute to epidemiological studies of PD in Asia, and may provide clues on risk factors for PD.

## Background

The population age structure is changing worldwide with many countries facing a rapidly ageing population [1]. As the number of older individuals within a population increases, so does the impact of chronic diseases on public health. Parkinson’s disease (PD) is one of the most common and complex neurodegenerative disorders that primarily affects older adults [2]. PD is characterized not only by classical parkinsonian motor symptoms, but also by a range of non-motor features including autonomic, cognitive, psychiatric, and behavioral dysfunctions [3]. Patients can survive up to a decade after diagnosis, but as PD progresses most patients experience severe physical and mental disabilities which impair their ability to live independently [4]. In addition, the medical cost of PD is one of the highest among neurological diseases [5, 6]. Thus, this disease poses an increasingly high physical, emotional, social, and economic burden on patients, their families and countries [5, 7]. In the face of rapidly aging societies, reliable epidemiologic data are urgently needed to provide insight into the risk factors, protective factors, and natural history of PD, as well as providing important information on the burden of this disease on the population.

Despite many epidemiological studies conducted over the years, many key aspects of this disease remain unclear. The estimated incidence and prevalence of PD reported in previous studies vary to a large extent due to difference in methodologies, diagnostic criteria, case-finding strategies and a limited sample size [2, 8]. Temporal trend studies are particularly rare due to difficulties in the consistent identification of PD patients in a stable and generalized population over time. Previous studies have reported that the incidence of PD increased [9, 10], stabilized [11], or slightly decreased [12–14] over the years.

The lack of appropriate and detailed epidemiological data is a barrier for the projection of future disease burden and effective public health planning. Reliable estimates of the prevalence and incidence of PD are needed for more effective prevention, diagnosis, and management. Until recently, most epidemiological studies of PD were conducted in Western countries [9, 12, 13, 15–18]. The epidemiology of PD in Asian countries is not well understood, and the comparison of trends in the incidence of PD is limited by a small number of studies with varying diagnostic criteria [8]. The epidemiology of PD is known to be different between the East and the West due to differences in genetics, environmental factors, and the denominator population [3, 8]. Therefore, further research is needed to study the incidence and prevalence of PD, particularly in Asia.

In this study, we investigated the temporal trends in the prevalence and incidence of PD in Korea over 6 years using data from the entire Korean population. The Korean government established a population-based registry database called the rare intractable diseases (RID) registration program, which includes PD. In the RID registry, all patients are registered after being diagnosed based on uniform diagnostic criteria that are defined by the government. Thus, using the RID database which is also linked with national health insurance data allows us to reliably examine unbiased nationwide epidemiological characteristics of PD.

## Methods

### Data source

The data used in this study were extracted from the national health insurance service (NHIS) database and RID registry. The NHIS is a mandatory universal health insurance system that covers the entire Korean population [19]. Each medical institution electronically submits all inpatient and outpatient health care utilization data to the NHIS for reimbursement purposes. The NHIS has a registration program for 138 RIDs, including PD, and offers co-payment reductions to patients for disease-related expenditure.

All patients with RIDs are required to have their diagnosis certified by a physician through the uniform diagnostic criteria that are distributed by the NHIS. After the physician’s assessment, the institution also reviews the diagnosis before submitting it to the NHIS. This systematic process ensures that the data regarding RIDs are reliable. Our study was based on data collected from all patients with PD in the NHIS-RID database between January 2010 and December 2015.

### Patient selection and diagnostic criteria

We selected all PD patients registered in the RID program during the 6-year study period. The diagnostic criteria for PD established by the NHI in the RID program are similar to the UK PD society brain bank clinical diagnostic criteria, and are as follows: 1) diagnosis of Parkinsonian syndrome (Parkinsonism): mild or worse bradykinesia and at least one of the following: muscular rigidity, rest tremor, postural instability; 2) the exclusion criteria for PD: history of strokes, head injury, definite encephalitis, drug side effects, and hypoxia; 3) supportive prospective positive criteria for PD: three or more required for diagnosis of definite PD in combination with step one: unilateral onset, rest tremor present, progressive disorder, persistent asymmetry affecting the side of onset most, excellent response (70–100%) to levodopa, severe levodopa-induced chorea, levodopa response for 5 years or more, clinical course of 10 years or more.

The database did not contain any personal identifiers as all identifiable personal information in the database was removed to comply with the privacy rules of the health insurance portability and accountability act. Informed consent was not required for this study as all the data was obtained from medical records. This study was performed based on the ethical principles of the Declaration of Helsinki of the World Medical Association. All procedural and ethical aspects of this study were approved by the Institutional Review Board of Korea University Ansan Hospital (IRB number: 2017AS0014).

### Statistical analysis

An incident case was defined as a newly diagnosed patient who was registered in the RID program as a PD patient during a given year. When defining an incident case, we excluded patients who were diagnosed with PD within 4 years before their registration in the RID program. The annual incidence was calculated by dividing the total number of incident cases by the total number of individuals in the entire population on December 31 of each year. These rates were directly standardized by age and gender to the total Korean population from the 2010 census. Age and gender specific incidences were calculated by dividing the number of incident cases in each age or gender group by the corresponding age or gender specific population and was expressed as cases per 100,000 individuals.

We defined a prevalent case as a patient registered with PD in the NHIS-RID database. When the NHIS registers the number of prevalent cases in the NHIS-RID database, it includes those who were registered as incident cases in the previous year, but does not include the number of patients who died or were disqualified (prison, military and immigration) in the previous year. In addition, prevalent cases of a year include all new patients registered with PD during a given year.

Age and gender specific prevalence was also calculated by dividing the number of prevalent cases in each age or gender group by the corresponding age or gender specific population and was expressed as cases per 100,000 individuals.

We also analyzed the incidence and prevalence of PD by metropolitan cities and provinces. In 2015, the administrative districts of the Republic of Korea consisted of eight metropolitan cities (Seoul, Busan, Daegu, Incheon, Gwangju, Daejeon, Ulsan and Sejong) and nine provinces (Gyeonggi, Gangwon, Chungbuk, Chungnam, Jeonbuk, Jeonnam, Gyeongbuk, Gyeongnam and Jeju).

## Results

### Incidence

**Fig. 1:**
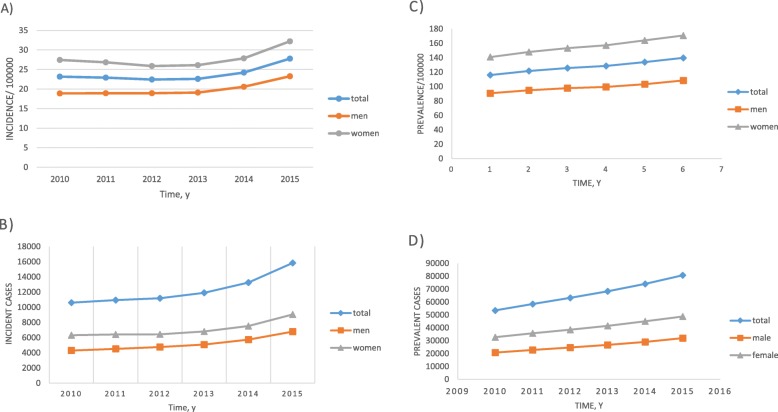
Trends in the incidence and prevalence of Parkinson’s disease (PD) in Korea, 2010–2015. **a** Incidence of PD, **b** incident cases of PD, **c** prevalence of PD, **d** prevalent cases of PD.

**Fig. 2:**
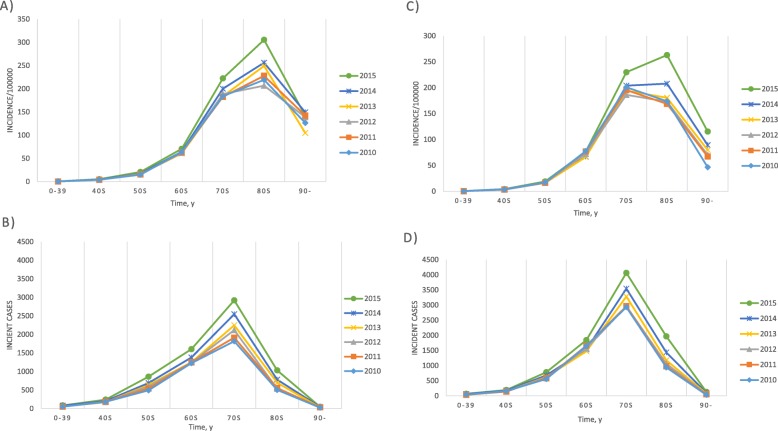
Incidence of Parkinson’s disease according to age and gender in Korea, 2010–2015. **a** Incidence in men, **b** incident cases in men, **c** incidence in women, **d** incident cases in women

The annual incident cases, incidence, and age or gender specific incidences of PD are displayed in Table 1 and Fig. 1a and b. Between 2010 and 2015, 73,726 new PD patients were registered (31,164 men and 42,562 women). The annual incidence of PD ranged from 22.4 to 27.8 cases per 100,000 individuals. The age- and gender-standardized incidence of PD was highest in 2015 in both men and women. The standardized incidence of PD in men increased over the study period. In women, the standardized incidence of PD was relatively constant until 2013, then increased significantly in 2014. There was a steady increase in the number of incident cases in both men and women (Fig. 2b, d). The incidence among men and women was 20.0 cases per 100,000 individuals and 27.7 cases per 100,000 individuals, respectively, with a female-to-male ratio of 1.4:1. Among men, the age group with the highest incidence of PD was between 80 and 89 years (Fig. 2a). In women, the highest incidence of PD was observed in individuals aged 70–79 years during 2010–2013 and in those aged 80–89 years from 2014 to 2015 (Fig. 2c). For both men and women, the incidence rate decreased in individuals older than 90 years.

**Table 1:** Incidence and prevalence of Parkinson’s disease in Korea, 2010–2015

Year	Total number of individuals (*10^3^)	Number of incident cases			Incidence per 10^5^ individualsª			Number of prevalent cases			Prevalence per 10^5^ individualsª		
	Total	Total	Male	Female	Total	Male	Female	Total	Male	Female	Total	Male	Female
2010	50,087	10,606	4294	6312	23.2	18.9	27.4	53,519	20,789	32,730	115.9	90.7	140.8
2011	50,362	10,940	4520	6420	22.9	18.9	26.8	58,504	22,789	35,715	121.5	94.7	147.8
2012	50,675	11,192	4755	6437	22.4	19.0	25.9	63,257	24,718	38,539	125.7	97.8	153.3
2013	50,922	11,897	5083	6814	22.6	19.1	26.1	68,292	26,758	41,534	128.5	99.5	157.1
2014	51,187	13,253	5725	7528	24.2	20.6	27.8	74,082	29,051	45,031	133.9	103.3	164.1
2015	51,473	15,838	6787	9051	27.8	23.3	32.2	80,747	32,002	48,745	139.8	108.4	170.8

^a^ Age- and sex- adjusted to 2010 population

### Prevalence

**Fig. 3:**
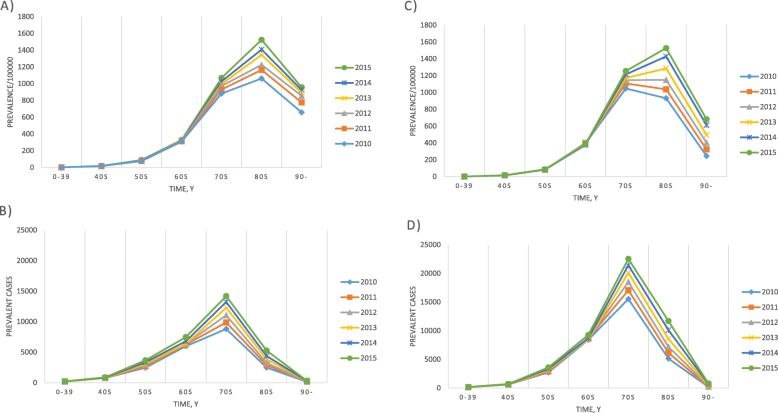
Prevalence of Parkinson’s disease according to age and gender in Korea, 2010–2015. **a** Prevalence in men, **b** prevalent cases in men, **c** prevalence in women, **d** prevalent cases in women

Data on the annual prevalence of PD is shown in Table 1, Fig. 1c and d. The number of prevalent PD cases increased from 53,519 in 2010 to 80,747 in 2015, of which 32,002 and 48,745 patients were men and women, respectively. The age- and gender-standardized prevalence of PD showed a gradual increase in both genders as the years progressed from 115.9 cases per 100,000 individuals in 2010 to 139.8 cases per 100,000 individuals in 2015. The standardized prevalence in 2015 was higher in women than in men, with 108.4 cases per 100,000 men and 170.8 cases per 100,000 women with a female-to-male ratio of 1.6:1. In men, the prevalence gradually increased with age and peaked at the age of 80–89 years (Fig. 3a). For women, the peak prevalence was reached in their 70s in 2010–2011 and in their 80s from 2012 onwards (Fig. 3c).

### Age-specific analysis

Table 2 shows the prevalence and incidence of PD in the individuals under or over 50 years of age. PD is relatively uncommon in individuals below age 50 and is classified as young onset Parkinson’s disease when it occurs before the age of 50 [20]. The incidence of PD increased in individuals under age 50 years from 1.2 cases per 100,000 individuals in 2010 to 1.7 cases per 100,00 individuals in 2015. The prevalence of PD in this age group also increased from 4.6 cases per 100,00 individuals in 2010 to 5.8 cases per 100,00 individuals in 2015. The prevalence of PD in the younger age group was higher in men than in women. Under the age of 50, the female-to-male ratio of the incidence and prevalence of PD in 2015 were 0.9: 1 and 0.8: 1, respectively.

**Table 2:** Incidence and prevalence of Parkinson’s disease (PD) according to age in Korea, 2010–2015

	Incidence						Prevalence					
	2010	2011	2012	2013	2014	2015	2010	2011	2012	2013	2014	2015
0–49 years												
Total												
Cases	435	422	412	403	520	590	1663	1663	1643	1680	1826	1974
Rate^a^	1.2	1.2	1.2	1.2	1.5	1.7	4.6	4.7	4.6	4.8	5.3	5.8
Male												
Cases	234	238	232	224	282	326	929	939	926	950	1023	1104
Rate^a^	1.3	1.3	1.3	1.2	1.6	1.8	5	5.1	5.1	5.3	5.7	6.2
Female												
Cases	201	184	180	179	238	264	734	724	717	730	803	870
Rate^a^	1.1	1.1	1.1	1.1	1.4	1.6	4.2	4.2	4.2	4.3	4.8	5.2
> 50 years												
Total												
Cases	10,171	10,518	10,780	11,494	12,733	15,248	51,856	56,841	61,614	66,612	72,256	78,773
Rate^a^	73.2	72	70.3	71.9	76.9	88.7	371.3	386.9	399.9	414.4	433.9	455.7
Male												
Cases	4060	4282	4523	4859	5443	6461	19,860	21,850	23,792	25,808	28,028	30,898
Rate^a^	63.1	63.1	63.4	65.1	70.2	80.1	307.5	320.7	332	344.1	359.8	381.3
Female												
Cases	6111	6236	6257	6635	7290	8787	31,996	34,991	37,822	40,804	44,228	47,875
Rate^a^	81.9	79.7	76.4	77.9	82.8	96.4	426.1	444.2	458.9	475.9	499	521.4

^a^Rate = Cases/ Population of that age group *100,000

In individuals aged 50 and older, the incidence of PD increased from 73.2 cases per 100,00 individuals in 2010 to 88.7 cases per 100,00 individuals in 2015. The prevalence of PD in this age group increased from 371.3 cases per 100,00 individuals in 2010 to 455.7 cases per 100,00 individuals in 2015. In this older age group, the incidence and prevalence of PD were higher in women than in men, and the female-to-male ratios in 2015 were 1.2: 1 and 1.4: 1, respectively.

### Regional analysis of trends

**Fig. 4:**
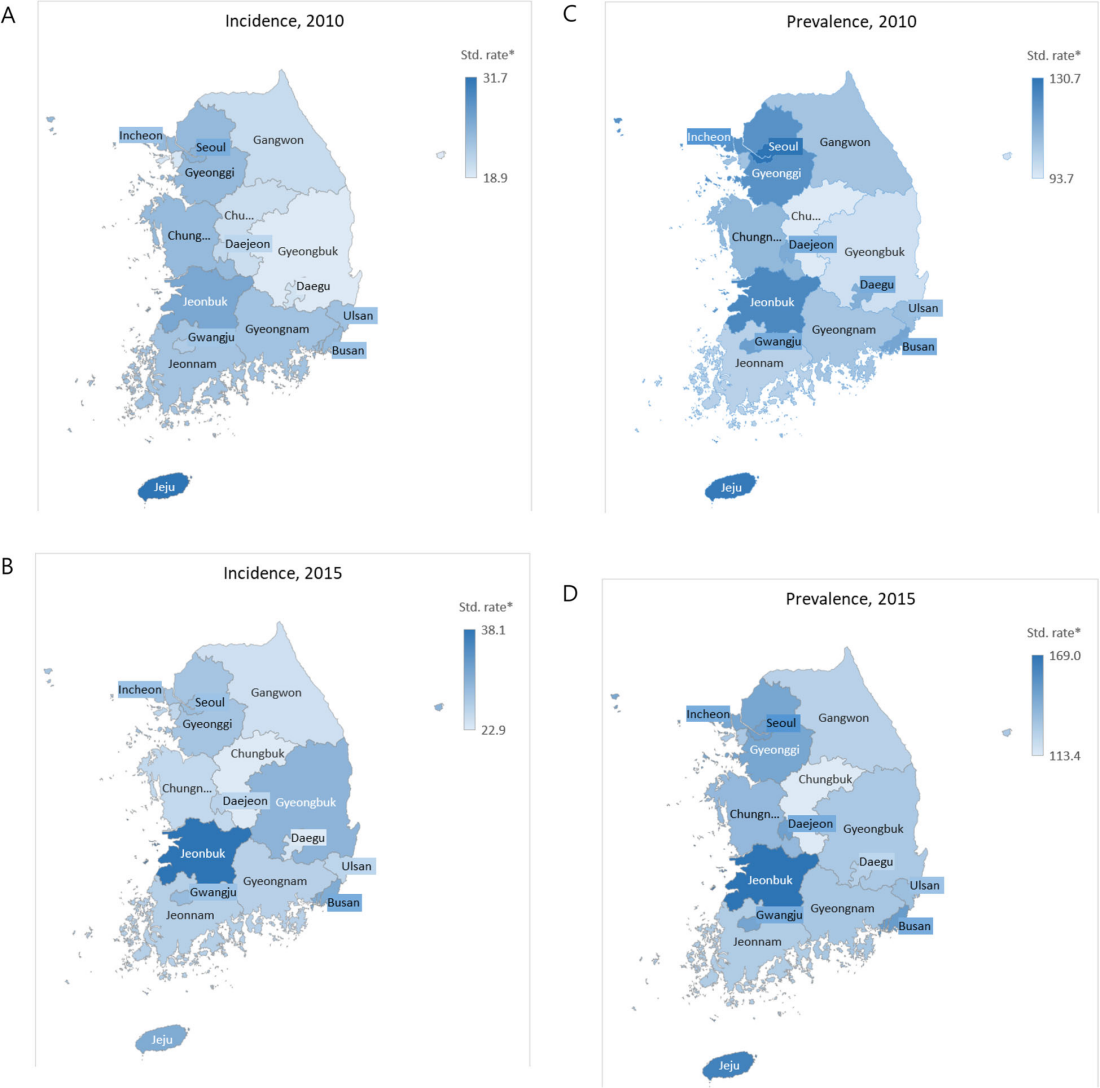
Regional trends in age- and sex-standardized incidence and prevalence per 100,000 population from 2010 to 2015. **a** Incidence in 2010, **b** Incidence in 2015, **c** Prevalence in 2010, **d** Prevalence in 2015. (The maps depicted in Figure 4 were produced by Joo-Hyun, Park.)

Table 3 and Fig. 4 show the trends in age-and sex-standardized incidence and prevalence of PD per 100,00 individuals by 17 regions from 2010 to 2015. During this period, the age-and sex-standardized incidence of PD increased in all regions of the country except for Sejong-si. Sejong-si was separated from provinces Chungbuk and Chungnam as a new administrative metropolitan city in 2012. Gyeongbuk and Jeonbuk experienced the largest increase in the incidence of PD from 2010 to 2015. The incidence ratios (2010 to 2015) of these two regions were 1.6 and 1.5, respectively. The highest incidence of PD was recorded in Jeju (31.7 cases per 100,00 individuals) in 2010 and Jeonbuk (38.1 cases per 100,00 individuals) in 2015.

**Table 3:** Age-and sex-standardized incidence and prevalence per 100,000 population by region from 2010 to 2015

	Incidence							Prevalence						
	2010	2011	2012	2013	2014	2015	Incidence ratio^a^ (2010–5)	2010	2011	2012	2013	2014	2015	Prevalence ratio^a^ (2010–5)
Seoul	24.9	22.0	22.0	23.2	24.6	28.7	1.2	130.7	132.5	137.0	138.2	143.0	148.6	1.1
Busan	24.1	22.8	22.6	25.5	27.7	30.9	1.3	115.6	124.9	131.2	134.7	142.6	150.2	1.3
Daegu	19.8	20.5	19.7	19.9	20.4	23.7	1.2	112.8	118.1	122.0	120.0	123.2	126.2	1.1
Incheon	19.4	18.5	17.8	20.4	21.6	25.5	1.3	106.3	112.8	113.5	117.9	122.8	129.5	1.2
Gwangju	22.3	23.9	21.1	19.7	24.5	28.9	1.3	117.7	132.9	134.2	132.8	140.8	146.5	1.2
Daejeon	21.0	26.8	27.8	26.6	22.5	26.0	1.2	113.9	128.3	140.0	144.2	147.3	147.9	1.3
Ulsan	23.4	20.8	17.4	21.4	24.2	25.7	1.1	107.5	113.0	118.9	123.6	129.5	133.7	1.2
Sejong	.	.	.	24.2	20.8	14.5	0.6	.	.	.	124.9	135.9	133.3	1.1
Gyeonggi	24.2	24.7	23.2	23.1	24.4	27.8	1.1	121.7	130.0	134.9	136.8	141.4	145.5	1.2
Gangwon	20.6	21.5	17.4	23.1	27.2	24.3	1.2	105.9	112.4	112.0	116.5	125.4	125.0	1.2
Chungbuk	20.3	21.6	17.9	20.1	18.3	22.9	1.1	93.7	104.2	102.2	105.4	108.2	113.4	1.2
Chungnam	24.1	23.6	24.3	22.4	24.7	25.3	1.0	109.5	118.5	124.7	126.5	132.7	136.7	1.2
Jeonbuk	26.2	24.5	27.8	28.4	32.9	38.1	1.5	124.4	127.1	136.8	143.3	153.5	169.0	1.4
Jeonnam	23.1	23.2	21.7	20.4	21.5	26.4	1.1	101.6	110.4	114.3	115.5	119.1	127.5	1.3
Gyeongbuk	18.9	21.2	20.9	21.0	23.0	29.8	1.6	97.1	103.0	110.2	113.1	119.0	129.4	1.3
Gyeongnam	23.3	23.0	21.0	22.2	23.9	26.2	1.1	105.6	112.7	115.2	121.1	125.5	130.5	1.2
Jeju	31.7	39.0	27.9	22.9	28.7	31.0	1.0	129.1	149.3	154.8	151.2	156.7	162.7	1.3

Rate = cases/the population of the region*100,000

^a^Incidence of 2015/incidence of 2010 or Prevalence of 2015/ prevalence of 2010

The age-and sex-standardized prevalence of PD increased in all regions from 2010 to 2015. The largest increase was recorded in Jeonbuk with a prevalence ratio of 1.4 from 2010 to 2015. The highest prevalence of PD was in Seoul in 2010 (130.7 cases per 100,00 individuals), and in Jeonbuk in 2015 (169.0 cases per 100,00 individuals). The region with the lowest prevalence of PD was Chungbuk. (93.7 cases per 100,00 individuals in 2010 and 113.4 cases per 100,00 individuals in 2015).

## Discussion

Our study was performed using recent population-based data encompassing the entire population of Korea. This is currently the only nationwide epidemiological study of PD in Korea. Furthermore, to the best of our knowledge, this is the first nationwide study that employed data from an entire population using uniform diagnostic criteria to assess recent temporal trends in the incidence and prevalence of PD in Asia.

Our results indicate an increasing trend in the annual prevalence and incidence rates of PD over the past 6 years. In our study, there were 16,152 new PD cases from 2010 to 2015, with a mean annual incidence of 23.9 cases per 100,000 individuals. The incidence of PD in men steadily increased throughout the study period. Conversely, the trend remained stable in women until 2013, then an increase in the incidence of PD was observed from 2014 onwards. The number of actual incident cases also gradually increased in both men and women. The lack of a clear trend in the incidence rate across the genders may be due to a difference in the denominator population caused by the growing aging population, especially among women.

Similar to our findings, several previous studies also reported an increase in the incidence of PD over time. In a 30-year study conducted in Olmsted County, Minnesota, Savica et al. [9] reported that the incidence of PD increased in men and remained stable in women. Only a few other studies evaluating trends in the incidence of PD have been conducted in the United Kingdom [13, 17], United States [9, 11], Netherlands [12], France [16], and Taiwan [10, 14]. These studies reported either a stable incidence [11, 16], a slight decrease [12–14, 17], or an increase [9, 10] in the incidence of PD over time. However, most of these studies [10, 11, 13, 14, 16] identified patients with PD through algorithmic searches of administrative databases without using reliable uniform diagnostic criteria. There is a substantial potential for misclassification when using this approach, making the results of these studies difficult to interpret.

The first possible cause for an increase in the incidence rate of PD is an increasingly older population. As PD is a neurodegenerative disease, increased incidence is expected in an ageing population [21]. The second possible explanation is an increased awareness of PD in patients and physicians as well as the availability of co-payments through the RID program. This leads to the diagnosis of previously undiagnosed cases and swifter referrals of patients with PD. In other words, there is a growing willingness to consult doctors regarding symptoms that were previously considered a normal part of aging which increases the likelihood that patients are examined by a neurologist. However, given the different trends observed in men and women and our consistent patient identification strategy, the observed increase is likely to be a real increase in incidence and prevalence.

We observed a gradual increase in the prevalence of PD throughout the study period. Similar results have also been reported in studies conducted in Japan [22], Taiwan [10, 14], and France [16] in the past decade. Both changes in the incidence of PD over time and the survival time of individuals with PD affect the prevalence of PD [23].

Interestingly, the present study demonstrated a preponderance in the prevalence and incidence of PD in the female population. Previous studies conducted on Western populations seem to indicate that PD is predominant in males [9, 13, 17, 18]. However, many Asian studies have reported female preponderance in PD [22, 24–28], while only some studies showed only male preponderance [14], or no difference between the genders [10, 29]. The reasons for this discrepancy are unclear and may be the results of several compounding factors. First, Asian individuals have different genetic susceptibilities and experience different environmental factors. Second, Asian women may face more risk factors, such as pesticide use, head trauma, agricultural occupations, toxic exposure, dietary deficiencies, and well-water drinking. Alternatively Asian women may also have fewer preventing factors, such as smoking, coffee drinking, and alcohol consumption compared to Western women [3]. Third, the ageing female population is increasing more rapidly than the male population [1]. Finally, men are more likely to be underdiagnosed because they have more competing diseases than women.

The incidence and prevalence of PD were higher in women than in men in patients over the age of 50, and higher in men than in women in patients with young onset PD. The neuroprotective effect of estrogen [30] is speculated to be the reason for the higher incident rate in male patients with young onset PD. However, the protective effects of estrogen decrease after menopause likely causing the observed increase in the incidence and prevalence of PD in the aging post-menopausal female population.

PD rarely developed before 50 years old, and the incidence increased sharply in patients in their 60s. The number of PD cases was highest in both male and female patients in their 70s during the entire study period. In the male population, the prevalence and incidence of PD consistently peaked between 80 and 89 years. In the female population, the peak prevalence and incidence of PD was between 70 and 79 years in 2010 and then increased to 80 and 89 years between 2012 and 2014. Any positive changes made in environmental risk factors in women seem to delay the onset of PD.

From 2010 to 2015, the prevalence of PD increased in all regions of the country. The incidence of PD also increased in all regions except for Sejong-si (a new administrative metropolitan city separated in 2012). In 2015, the highest incidence and prevalence of PD was in Jeonbuk province. These findings can be utilized by policy makers to prioritize advancements in national health.

This study has certain strengths. First, this is a nationwide study that surveyed the entire population of Korea for 6 years. This nationwide evaluation of the epidemiological features of PD provides a better understanding of the national patterns of PD epidemiology in South Korean patients with a recent diagnosis of PD. This study had no selection bias as it examined a stable and generalized population. Second, the epidemiological data in this study is more reliable than the data reported in previous studies because clear and uniform diagnostic criteria were used. Most previous nationwide studies used algorithmic searches of administrative databases to diagnose PD patients leading to potential misclassification depending on the different algorithms used. To the best of our knowledge, this study is the first Asian study to examine long-term trends in the incidence of PD using uniform and reliable diagnostic criteria.

This study also has a few limitations. First, because we used insurance claims data, we could not rule out the possibility of the misclassification of patients with PD. However, the diagnosis of PD in the RID database is based on diagnostic criteria standardized in hospitals by the NHI. Before being submitted to the NHI, the physician’s diagnosis passes through a verification process and is reviewed by another health care personnel in their institution. Through this process, we are certain that a diagnosis in the RID database has a relatively higher accuracy than other insurance data. This is also confirmed by other epidemiological studies of RID, in which a high agreement has been reported between hospital and administration data [31, 32]. Although neuropathological assessment is the gold standard for diagnosis of PD, there are no generally accepted standard pathological diagnostic criteria for PD [33], and the diagnosis of PD relies on clinical examination [3, 34]. Second, we could not identify the stage of PD in this study. Third, the diagnosis of PD is likely to be underestimated because of low hospital utilization or other comorbid diseases in older patients [15]. There may be an unknown number of individuals with PD who were not accurately diagnosed because of inadequate facilities and staffing in areas of low socioeconomic status. Although other studies have used several methods to calculate the number of patients with PD in a community, there is no gold standard and each method has its own limitations [35, 36]. Fourth, we could not identify risk factors because we were unable to access the underlying disease information of PD patients. Therefore, we were unable to directly examine whether changes in environmental risk factors, such as a decreased prevalence of smoking, were responsible for the observed increase in the incidence of PD. Finally, the number of prevalent cases in that year excluded not only the number of patients who died, but also the number of people who have become disqualified (prison, military, immigration, etc.) in the previous year. Currently, the NHIS-RID database does not provide information on insurance disqualification. Hence, the observed prevalence may be affected by the number of deaths and the number of people disqualified each year. However, the difference caused by this discrepancy will not be large. The results of this study will contribute greatly to further research by presenting precise macroscopic epidemiologic data.

## Conclusion

In Korea, the prevalence and incidence of PD has gradually increased in recent years. Over this period, PD occurred more in women than in men. This study will aid the assessment of disease burden and resource allocation in PD. Furthermore, a thorough analysis of this trend may provide clues on the environmental factors involved in the etiology and characteristics of PD in Korea. To acquire a better understanding on the effects of multiple potential risk factors on PD, in-depth studies on the role of genetic and environmental factors in the epidemiology of PD are required.

## Data Availability

Data is available from the Korea National Health Insurance Sharing Service Institutional Data Access / Ethics Committee (https://nhiss.nhis.or.kr/bd/ay/bdaya001iv.do) for researchers who meet the criteria for access to confidential data. Researchers can apply for the National Health Insurance data sharing service upon approval by the Institutional Review Board of their institution. After a review by the Korea National Health Insurance Sharing Service Institutional Data Access / Ethics Committee, authors are required to pay a data access fee and confirm that other researchers will be able to access the data in the same manner as the authors.

## Abbreviations

NHIS: National health insurance service
PD: Parkinson’s disease
RID: Rare intractable disease
